# Proton Pump Inhibitors Exert Anti-Allergic Effects by Reducing TCTP Secretion

**DOI:** 10.1371/journal.pone.0005732

**Published:** 2009-06-01

**Authors:** Sunghee Choi, Hyun Jung Min, Miyoung Kim, Eun Sook Hwang, Kyunglim Lee

**Affiliations:** College of Pharmacy, Center for Cell Signaling & Drug Discovery Research, Ewha Womans University, Seoul, Korea; Centre de Recherche Public de la Santé (CRP-Santé), Luxembourg

## Abstract

**Background:**

Extracellular translationally controlled tumor protein (TCTP) is known to play a role in human allergic responses. TCTP has been identified outside of macrophages, in activated mononuclear cells, and in biological fluids from allergic patients. Even TCTP devoid of signal sequences, is secreted to extracellular environment by an yet undefined mechanism. This study is aimed at understanding the mechanism of TCTP release and its regulation. A secondary goal is to see if inhibitors of TCTP release can serve as potential anti-allergic asthmatic drugs.

**Methodology/Principal Findings:**

Using Western blotting assay in HEK293 and U937 cells, we found that TCTP secretion is reduced by omeprazole and pantoprazole, both of which are proton pump inhibitors. We then transfected HEK293 cells with proton pump expression vectors to search for the effects of exogeneously overexpressed H^+^/K^+^-ATPase on the TCTP secretion. Based on these *in vitro* data we checked the in vivo effects of pantoprazole in a murine model of ovalbumin-induced allergy. Omeprazole and pantoprazole reduced TCTP secretion from HEK293 and U937 cells in a concentration-dependent fashion and the secretion of TCTP from HEK293 cells increased when they over-expressed H^+^/K^+^-ATPase. In a murine model of ovalbumin-induced allergy, pretreatment with pantoprazole reduced infiltration of inflammatory cells, increased goblet cells, and increased TCTP secretion induced by OVA challenge.

**Conclusion:**

Since Omeprazole and pantoprazole decrease the secretion of TCTP which is associated with the development of allergic reaction, they may have the potential to serve as anti-allergic (asthmatic) drugs.

## Introduction

Translationally controlled tumor protein (TCTP) is expressed in almost all mammalian tissues. Intracellular TCTP levels respond to various extracellular signals and agents such as growth factors, cytokines, and stress conditions [1]–[3]. Extracellular TCTP has also been reported to be present in the supernatants of human U937 macrophage cell cultures [4], outside of mononuclear cells and platelets, in nasal washings, skin blister fluids, and bronchoalveolar lavage (BAL) fluids during late allergic reactions [5]–[9]. Human recombinant TCTP stimulates the secretion of histamine, IL-4 and IL-13 from basophils. TCTP also causes chemotaxis and IL-8 secretion from eosinophils [10], [11].

Most secretory proteins have signal sequences composed of 13–30 hydrophobic amino acids at their N-termini. Some leaderless proteins, however, can exit from a cell in an ER-Golgi independent fashion, for example, interleukins 1α and 1β, FGF2, thioredoxin, lipocortin, galectin, HIV-tat protein, annexin, and vas deferens protein. TCTP, with no classical leader sequence that might explain its extracellular presence, belongs to these unusual proteins, which exit from a cell without passing through the classical secretion pathway [12], [13]. How these proteins are non-classically secreted from cell has not yet been defined. Contrary to an early perception, the selective release of the ‘leaderless’ proteins can be unequivocally distinguished from conventional ER-Golgi-mediated protein secretion which is not a consequence of impaired plasma membrane integrity or cell death. Several potential mechanisms were proposed for such unconventional protein secretion, involving: lysosomal and exosomal secretion, plasma membrane resident transporters, and membrane blebbing [14], but a definitive understanding of the secretion mechanism for leaderless proteins has not emerged.

Omeprazole is an active ingredient of Prilosec, used to treat peptic ulcer. It is a specific inhibitor of the human gastric H^+^/K^+^-ATPase [15], which at neutral pH, permeates cell membranes and accumulates in acidic cellular compartments, such as lysosomes, where it undergoes protonation. The protonated form becomes an active sulfenamide compound and acts as a potent proton pump inhibitor (PPI) [16]. Activated omeprazole was shown to inhibit human gastric H^+^/K^+^-ATPase and halt acid secretion by parietal cells [17]. Pantoprazole is a modified form of omeprazole and also is also a PPI. PPIs have also been shown to inhibit neutrophil functions [18] including adhesion to endothelial cells [19], [20], phagocytosis, acidification of phagolysosomes [21], and production of reactive oxygen intermediates [22].

Although the secretion of TCTP is well documented, how it is regulated is not clear. Because the factors contributing to its secretion and the underlying mechanisms are still unclear we tried various ATPase inhibitors (Na^+^/K^+^-ATPase, plasmamembrane H^+^/K^+^-ATPase, plasmamembrane Ca^2+^-ATPase) based on the report that FGF-2 release is related to Na^+^/K^+^-ATPase inhibitors [23]. We found that omeprazole and pantoprazole inhibit TCTP release in a concentration-dependent fashion *in vitro*. We confirmed this phenomenon *in vivo* and came to the conclusion that omeprazole and pantoprazole have the anti-allergic asthmatic potential by reducing TCTP secretion.

## Methods

### Antibodies

Mouse 12CA5 anti-HA monoclonal antibody, purified rabbit anti-GFP polyclonal antibody, mouse anti-Na^+^/K^+^-ATPase α1 monoclonal antibody (C464.6), and anti-flag® M2 monoclonal antibody were purchased from Zymed Laboratories Inc., InVitrogen, Upstate, and Sigma, respectively.

### Cell culture and cell secretion assays

HEK293 cells (ATCC) were cultured in Dulbecco's modified Eagle's media (DMEM; Invitrogen) containing 10% fetal bovine serum, 1% penicillin-streptomycin, 2 mM glutamine, and 20 mM HEPES. U937 cells (ATCC) were cultured in RPMI-1640 media (Invitrogen) containing 10% fetal bovine serum, 1% penicillin-streptomycin, and 10 mM HEPES. On the day of experiment, HEK293 cells were detached from culture dishes, using with trypsin-EDTA, and washed twice with serum-free media. U937 cells were collected by centrifugation and washed twice with serum-free media. The collected cells were stained with trypan blue, counted for live cells, and plated. The cells were resuspended in serum-free media and incubated for indicated times with or without omeprazole (Sigma) and pantoprazole (Byk Gulden, Germany) or ionomycin (Sigma). Cell viability was assessed by trypan blue exclusion or CCK-8 (Dojindo Molecular Technologies, Japan). At the end of incubation, supernatants were collected and centrifuged (300×g) for 5 min to remove non-adherent cells. The supernatants were recentrifuged (5,000×g) for 10 min to remove cell debris and nuclei and concentrated by centrifugation using vivaspin 500 (10,000 molecular weight cut-off; Sartorius, France), and resuspended in 4× SDS sample buffer. Each sample (indicated amount of total proteins was resolved by SDS-PAGE. Cells were solubilized in lysis buffer containing 1% Nonidet P-40, 50 mM Tris-HCl (pH 7.4), 150 mM NaCl, 1 mM EDTA, 0.25% deoxycholate, 2 mM sodium orthovanadate, 1 mM sodium fluoride and mixture of protease inhibitors. After transfer the membranes were routinely blotted.

### Plasmid construction

To construct the plasmid, human TCTP/p3XFLAG-CMV-14, human TCTP insert was made by PCR amplification using primers with *Bam*HI and *Eco*RI sites. Insert DNA and p3XFLAG-CMV-14 empty vector were excised by *Bam*HI and *Eco*RI restriction enzymes and sticky-end ligated. The plasmid was confirmed by DNA sequencing. To construct the plasmid, rat H^+^/K^+^-ATPase α1/pEGFP-N1, rat H^+^/K^+^-ATPase α1 insert was made by PCR amplification using primers with *Hind*III and *Xho*I sites. Insert DNA and pEGFP-N1 empty vector were excised by *Hind*III and *Xho*I restriction enzymes and sticky-end ligated. The plasmid was confirmed by DNA sequencing. Rat Na^+^/K^+^-ATPase β1/pcDNAI-neo was constructed as previously described [24].

### Transfections

HEK293 cells were plated and a day later, transfected by the Ca_2_PO_4_ precipitation method or WelFect EX PLUS reagent (WelGene, Korea). In triple transfections, the ratio of rat H^+^/K^+^-ATPase α1-GFP: HA-rat Na^+^/K^+^-ATPase β1: human TCTP-3X flag was 3∶1∶1. Secretion assays were performed after 2 days. In some experiments, omeprazole or pantoprazole was added during the secretion assay.

### Development of airway inflammation

Male BALB/c mice were purchased from the Central Lab. Animal Inc. (Korea), and were kept in our animal facility for at least 1 week before use. All animals were handled in strict accordance with good animal practice as defined by the relevant national and/or local animal welfare bodies, and all animal work was approved by Ewha Womans University's institutional animal care and use committee. The mice were sensitized on day 0, and on day 14, with intraperitoneal (i.p.) injection of OVA (Sigma, 50 µg per each) in alum (Thermo scientific). Starting on day 28, the sensitized mice were challenged with daily intranasal injections (with pipetman) of PBS or OVA (300 µg per each) for seven days. For pantoprazole treatment, the mice were pre-treated (i.p.) with indicated dose of Pantoloc containing pantoprazole as active ingredient; (Byk Gulden, Germany) 30 min prior to OVA challenge and sacrificed on day 37. Bronchoalveolar lavage lung fluids (BALF) and lungs were collected. Western blotting for TCTP detection was performed on BALF.

### Lung analyses

Lung tissues were fixed in 4% paraformaldehyde, dehydrated in alcohol, embedded in paraffin and cut into 5 µm thick slices. Tissue section was stained with PAS solution (Sigma). Images were acquired using optical microscope (Nikon Eclipse E200, Japan).

### Statistics

Data are expressed as mean±S.D. Significances of differences between two groups were determined using the unpaired student's t-test. Statistical significance was set at p<0.05.

## Results

### Omeprazole and pantoprazole inhibit TCTP secretion from U937 and HEK293 cells

**Figure 1:**
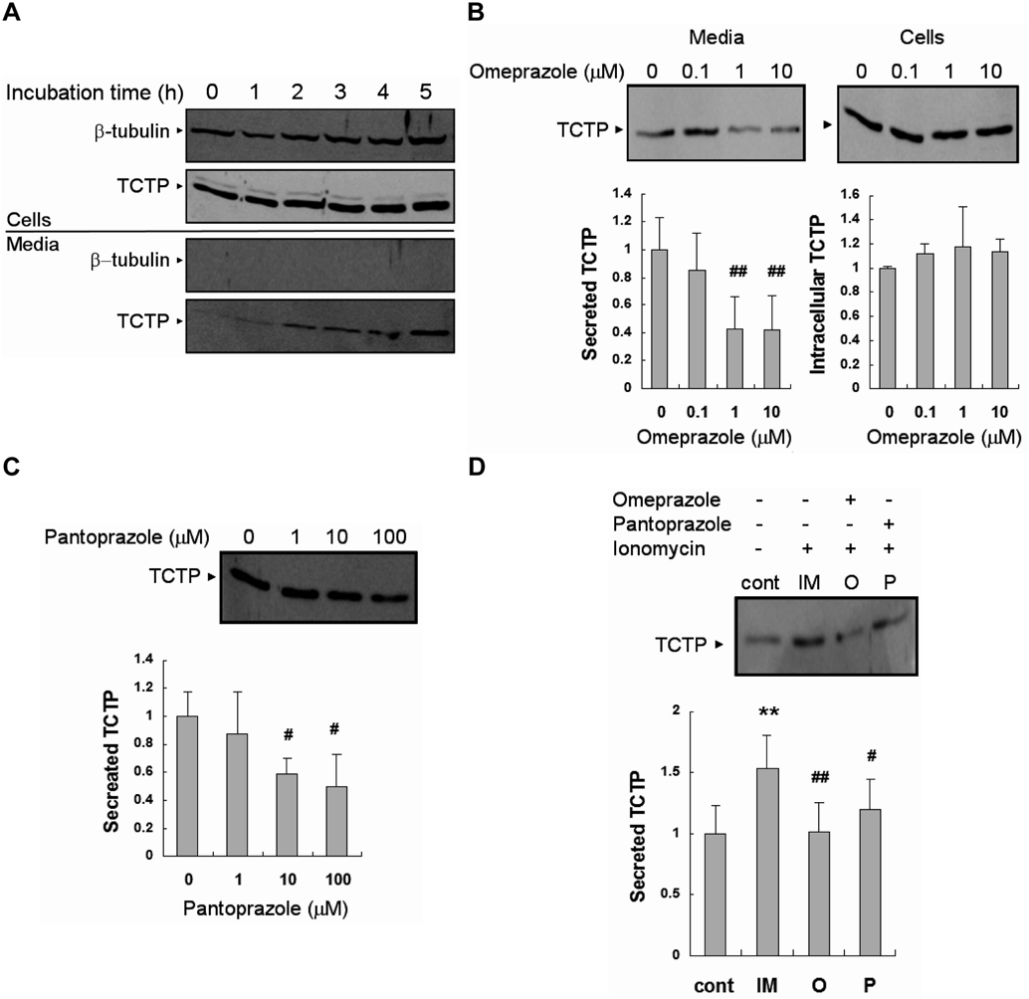
TCTP secretion, both constitutive and induced from U-937 cells is inhibited by omeprazole and pantoprazole. (A) TCTP secretion is increased with incubation time in serum-free conditioned media. β-tubulin is a loading control in cells, while it is a cell lysis marker in media. WB: anti-β-tubulin Ab (rabbit polyclonal antibody, H-235, Santa Cruz Biotechnology) and anti-TCTP Ab (rabbit polyclonal antiserum, generated using recombinant His-tagged TCTP as the immunogen), (B) Omeprazole treatment during incubation in conditioned media (5 h) is capable of decreasing of TCTP secretion in concentration-dependent manner. ‘O’ in the graph means omeprazole. WB: anti-TCTP Ab, (C) Pantoprazole treatment (5 h) inhibits TCTP secretion in concentration-dependent manner. ‘P’ in the graph means pantoprazole. WB: anti-TCTP Ab, (D) Both of omeprazole (O) and pantoprazole (P) pre-treatments reduce the TCTP secretion induced by ionomycin (IM) treatment (2 µM, 10 min). 1 µM omeprazole and 100 µM pantoprazole were used. Both chemicals were treated 1 h prior to ionomycin treatment. WB: anti-TCTP Ab. Data from panel B to panel D represent means±S.D. from three independent experiments. #: inhibition, p<0.05, vs. non-treated cells (panel B, C), vs. IM-treated cells (panel D), ##: inhibition, p<0.01, vs. non-treated cells (panel B, C), vs. IM-treated cells (panel D), **: increase, p<0.01, vs. non-treated cells.

**Figure 2:**
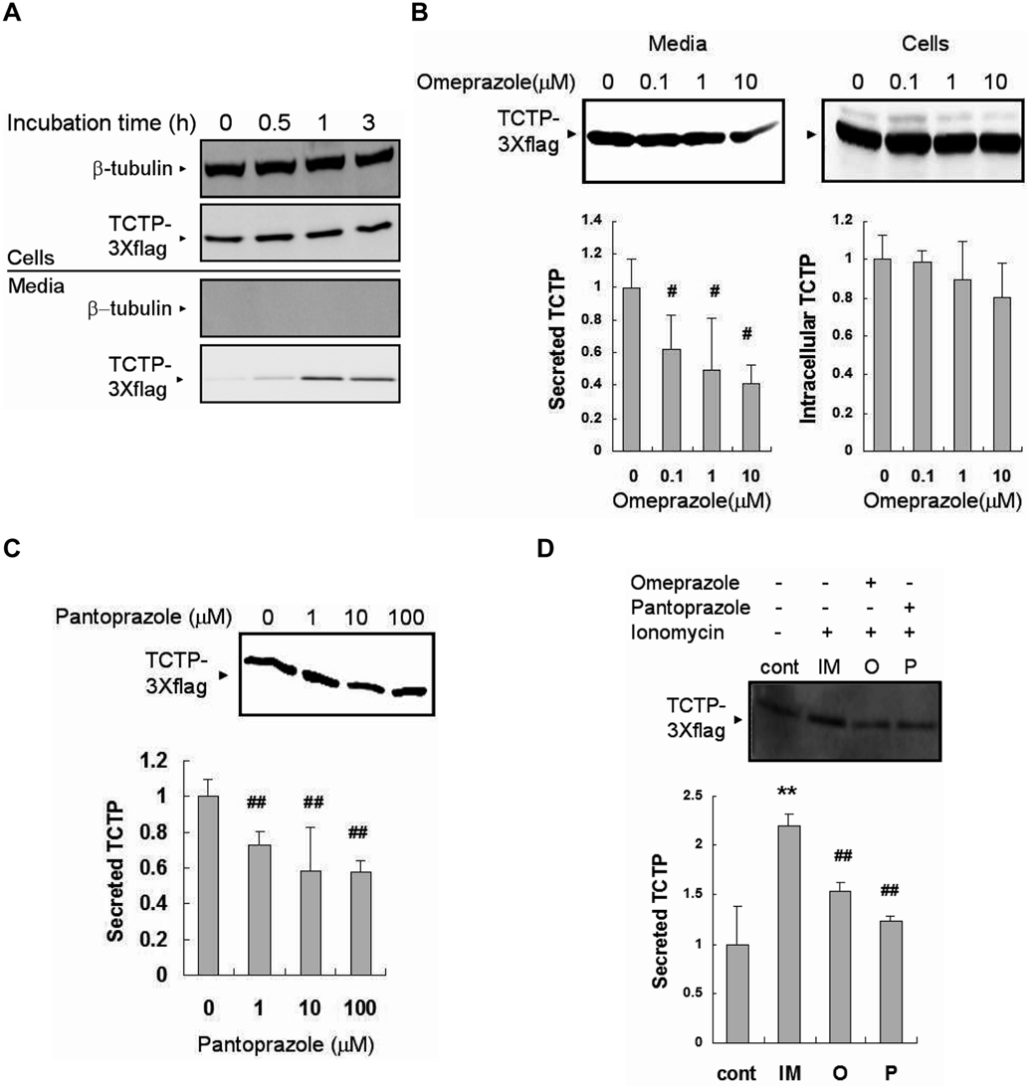
The secretion of exogeneously expressed TCTP in HEK293 cells is inhibited by omeprazole and pantoprazole. (A) TCTP secretion is increased with incubation time in serum-free conditioned media. β-tubulin is a loading control in cells, while it is a cell lysis marker in media. WB: anti-β-tubulin Ab and anti-flag Ab, (B) Omeprazole treatment during incubation in conditioned media (3 h) is capable of decreasing of TCTP secretion in concentration-dependent manner. ‘O’ in the graph means omeprazole. WB: anti-flag Ab, (C) Pantoprazole treatment (3 h) inhibits TCTP secretion in concentration-dependent manner. ‘P’ in the graph means pantoprazole. WB: anti-flag Ab, (D) Both of omeprazole (O) and pantoprazole (P) pre-treatments reduce the TCTP secretion induced ionomycin (IM) treatment (2 µM, 10 min). 1 µM omeprazole and 100 µM pantoprazole were used. Both chemicals were treated 1 h prior to ionomycin treatment. WB: anti-flag Ab. Data from panel B to panel D represent means±S.D. from three independent experiments. #: inhibition, p<0.05, vs. non-treated cells (panel B, C), vs. IM-treated cells (panel D), ##: inhibition, p<0.01, vs. non-treated cells (panel B, C), vs. IM-treated cells (panel D), **: increase, p<0.01, vs. non-treated cells.

We first established the incubation period needed for detection of secreted TCTP. Extracellular TCTP was first identified in the supernatants of U937 cells [4] and further studies on TCTP release were done using HEK293 cells. U937 cells were incubated in serum-free RPMI media for 0, 1, 2, 3, 4, or 5 h, and the collected supernatants and cells were processed, resolved by SDS-PAGE, and immunoblotted for TCTP. The secreted amount of TCTP increased with incubation time and reached maximum after 5 h incubation (Figure 1A). To ensure that TCTP resulted from an active secretion process rather than from contamination from cell lysis or non-specific release, the supernatants were also immunoblotted against β-tubulin, one of the abundant cytosolic proteins. Beta tubulin was clearly identified in cell lysates but not in the corresponding supernatants (Figure 1A). In HEK293 cells, it was difficult to detect the secretion of endogeneous TCTP. HEK293 cells were therefore transfected with TCTP-3Xflag and 2 days later placed in serum-free media and their supernatants were assayed for the presence of TCTP. Transfected HEK293 cells were incubated in serum-free DMEM media for 0, 0.5, 1, or 3 h, and the collected supernatants and cells were processed, and resolved by SDS-PAGE, and immunoblotted for TCTP-3Xflag. By 1 h, exogenously expressed TCTP was detected in HEK293 cell-derived supernatants; these levels were not further enhanced at 3 h. As with U937, there was no evidence of cell lysis or non-specific protein release (Figure 2A).

When the secretion assays were performed with serum-free media supplemented with omeprazole or pantoprazole, the amounts of TCTP detected in the conditioned media were reduced in a concentration dependent fashion. In U937 cells, 10 µM omeprazole reduced TCTP secretion by about 60% (Figure 1B) and by 50% with 100 µM pantoprazole (Figure 1C). In HEK293 cells, the reduction of TCTP secretion was 60% and 40% for omeprazole and pantoprazole, respectively (Figure 2B, 2C). In these experiments, we used omeprazole at lower concentration than pantoprazole for the following reasons. First, omeprazole showed cytotoxic effect at concentrations higher than 100 µM, probably due to the excessive inhibition of H^+^/K^+^-ATPase. Second, the reconstituted pantoprazole caused precipitation after freezing and thawing and precipitation causes some reduction in activity. To avoid such precipitations, it was dissolved as soon as it was reconstituted, and divided into small aliquots. The viability of the cells treated by omeprazole and pantoprazole was confirmed with trypan blue or CCK-8 kit; there was no significant difference between untreated and treated cells. The inhibition of TCTP secretion by omeprazole and pantoprazole was also seen in ionomycin (IM) treated cells. IM is a Ca^2+^ ionophore and induces secretion of many proteins in an ER-Golgi independent process. With U937 cells, IM treatment increased TCTP secretion by approximately 50% and pre-treatment with omeprazole and pantoprazole blocked this increase (Figure 1D). HEK293 cells transfected with TCTP-3Xflag also showed similar patterns (Figure 2D).

These results indicate that omeprazole and pantoprazole inhibit TCTP secretion, regardless of whether it is endogeneously or exogeneously expressed. Also secretion inducers had no effects on these phenomena.

### α-subunit of H^+^/K^+^-ATPase modulates TCTP export

**Figure 3:**
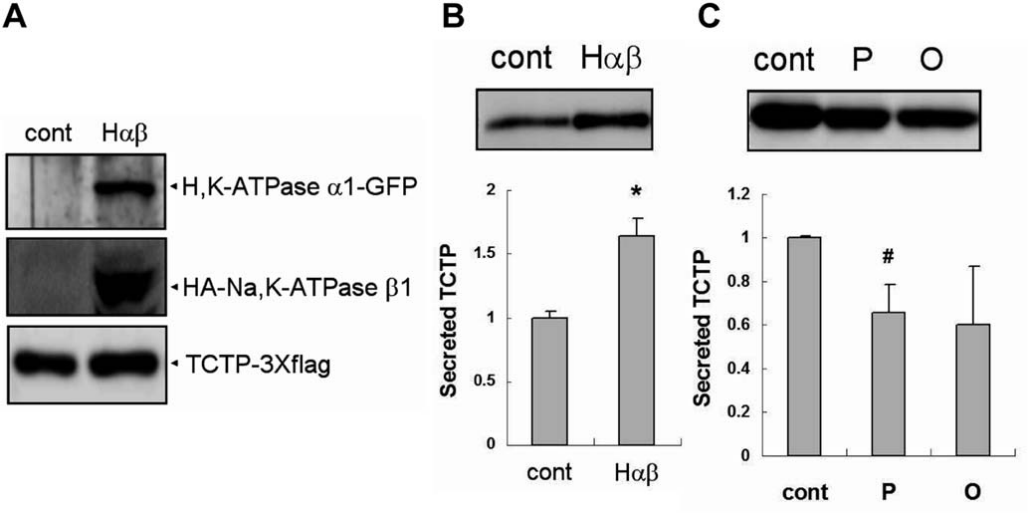
Increased TCTP secretion by over-expression of proton pump is inhibited by pantoprazole in HEK293. (A) Comparison of protein expressions in control and Hαβ samples. Control sample was transfected with two empty vectors (pEGFP-N1 and pcDNAI-neo) and TCTP-3Xflag construct. Hαβ sample was transfected with rat H^+^/K^+^-ATPase α1-GFP, HA-rat Na^+^/K^+^-ATPase β1, and TCTP-3Xflag constructs. WB: anti-GFP Ab (purified rabbit polyclonal antibody, InVitrogen), anti-HA Ab (mouse 12CA5 monoclonal antibody, Santa Cruz), and anti-flag Ab, (B) TCTP secretion is increased by over-expression of H^+^/K^+^-ATPase α1 and Na^+^/K^+^-ATPase β1. Both groups of cells were incubated for 3 h in conditioned media. WB: anti-flag Ab, (C) The secretion assay data for the cells transfected with H^+^/K^+^-ATPase α1, Na^+^/K^+^-ATPase β1, and TCTP. Pantoprazole (1 mM) or omeprazole (1 mM) was treated during 3 h secretion assay. ‘O’ in the graph means omeprazole and ‘P’ does pantoprazole. WB: anti-flag Ab. Data of panel B and panel C represent means±S.D. from three independent experiments. #: inhibition, p<0.05, vs. non-treated cells, *: increase, p<0.05, vs. the cells transfected with two empty vectors (pEGFP-N1 and pcDNAI-neo) and TCTP-3Xflag construct.

The major effect of PPIs is to inhibit the ion transport activity of H^+^/K^+^-ATPase by binding to the catalytic α-subunit. Since omeprazole and pantoprazole inhibited TCTP export, we investigated whether changes in α-subunit level of H^+^/K^+^-ATPase affect TCTP export. HEK293 cells were co-transfected with plasmid expression vectors encoding TCTP, rat α1-subunit of H^+^/K^+^-ATPase, and rat β1-subunit of Na^+^/K^+^-ATPase. As shown in Figure 3B, co-overexpression of α1 and β1 with TCTP elevated TCTP secretion by over 60%, compared with control cells expressing TCTP only. This increase was not seen in the cells transfected with β1 and TCTP except α1. This suggests that the complex of α1-subunit and β1-subunit is in some way involved in TCTP export. TCTP secretion induced by co-expression of α1 and β1 was reduced by pre-treatment of omeprazole and pantoprazole (Figure 3C). But only pantoprazole had a statistically significant effect. Since the concentration of omeprazole and pantoprazole used in Figure 2 was not sufficient to reverse the increase of TCTP release, a tenfold concentration was used. Cell viability was not changed by these pre-treatments.

### Effect of pantoprazole on the TCTP levels in BAL fluids from OVA-sensitized mice

**Figure 4:**
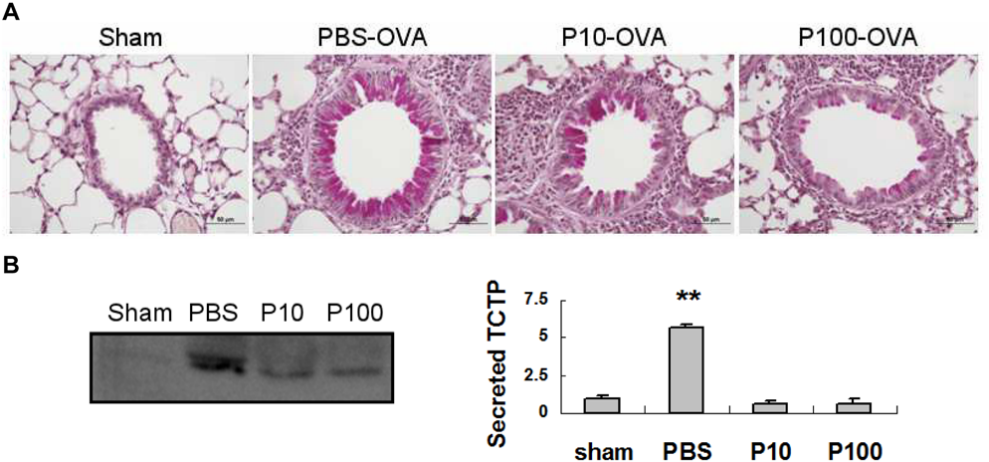
Pantoprazole alleviates asthmatic responses and inhibits TCTP secretion in OVA-sensitized/challenged mice. (A) PAS staining results of lung sections. OVA-sensitized/challenged mice show marked increase of the mucus-producing granular goblet cells in the bronchial alveoli relative to sham mice. The increased PAS positive goblet cells in OVA challenged mice were reduced by pantoprazole pre-treatment in amount dependent manner. Pantoprazole pre-treatment also reduced marked infiltration of the airway wall by PAS positive inflammatory cells, (B) Western blotting data using BALF of allergic mouse model. WB: anti-TCTP antibody. Mice were sensitized with OVA and challenged with PBS or OVA five times every other day. ‘P’ means pantoprazole and indicated numbers (10 and 100) mean dosage. #: inhibition, p<0.05, vs. OVA challenged but no pre-treatment, ##: inhibition, p<0.01, vs. OVA challenged but no pre-treatment, **: increase, p<0.01, vs. sham group. sham: negative control, OVA sensitized but not challenged, n = 4. OVA: positive control, OVA sensitized and challenged with no pre-treatment, n = 5. P 10: OVA sensitized and challenged with pantoprazole 10 mg/kg pre-treatment (30 min), n = 6. P 100: OVA sensitized and challenged with pantoprazole 100 mg/kg pre-treatment (30 min), n = 6.

It has been shown that TCTP appears in biological fluids during the late allergic reaction [5]–[9]. We used the OVA-challenged mice for examining the effect of pantoprazole *in vivo*. Periodic Acid-Schiff (PAS) staining of lung sections showed a marked increase in mucus-producing granular goblet cells in the proximal airways of OVA-sensitized/challenged mice relative to saline-treated control mice (Figure 4A). The increase of PAS positive goblet cells in mice were reduced by pretreatment with pantoprazole in a dose dependent manner. Pantoprazole pre-treatment also reduced markedly, the infiltration of the airway wall by PAS positive inflammatory cells. Increase of mucus-producing goblet cells and inflammatory cell infiltration is a prominent histopathological feature of the murine asthmatic lung.

Next, we examined the changes in TCTP secretion into BAL fluids. As shown in Figure 4B, OVA challenged mice showed increased TCTP in BAL fluids. Similar to *in vitro* data, pantoprazole pre-treatment inhibited TCTP secretion to that present in sham group. These results indicate that pantoprazole might exert its anti-allergic effect by blocking TCTP secretion.

## Discussion

TCTP is known to be a secreted protein, but the factors contributing to its secretion and the underlying mechanisms are still unclear. There are many studies about the secretion mechanisms of the other non-typically secreted proteinsother than TCTP. Secretion of the catalytic subunit of Na^+^/K^+^-ATPase is reported to be related to fibroblast growth factor-2 (FGF-2). In this study, the authors searched for some small molecules that might cause specific perturbation of the plasma membrane and interfere with FGF-2 release. They assumed that there is a plasma membrane translocation apparatus (PMTA) through which FGF-2 is exported. They found cardenolides like ouabain inhibit FGF-2 secretion and that Na^+^/K^+^-ATPase acts as a PMTA component or regulator. Based on this report, we searched for the inhibitors that act on plasma membrane and inhibit TCTP secretion. Eventually we found that proton pump inhibitors (PPIs) such as omeprazole and pantoprazole decrease the leaderless TCTP export. Possible cytotoxic effects of omeprazole and pantoprazole could be ruled out, since the trypan blue or CCK-8 assay showed no toxic effects when these drugs were used. Also, reduction in TCTP secretion by PPIs did not result from altered protein synthesis or increased instability of intracellular pools, because the treatment with these drugs did not modify the amounts of the intracellular proteins remarkably (Figs. 1B, 2B).

Omeprazole is used to treat peptic ulcer because it is a specific inhibitor of the human gastric H^+^/K^+^-ATPase. It is a proton pump inhibitor (PPI). Other Chemicals containing pantoprazole, rabeprazole, lansoprazole, and esomeprazole are also PPIs. PPIs interact with the catalytic α-subunit of H^+^/K^+^-ATPase and thereby inhibit its ability to exchange K^+^ for H^+^, hydrolyze ATP, and maintain an electrochemical gradient across the plasma membrane. In addition to reduction of gastric acid secretion through H^+^/K^+^-ATPase inhibition, various biological effects of omeprazole have been investigated, including antileishmanial (antimicrobial) activity [25], relaxation of human arteries [26], and inhibition of neutrophil chemotaxis [27]. Although the major target of PPIs is heretofore believed to be gastric H^+^/K^+^-ATPase (known to be mainly in parietal cells of stomach), other molecules, for example putative H^+^/K^+^-ATPase that are sensitive to PPIs and are unrelated to gastric H^+^/K^+^-ATPase or completely new targets other than proton pump, might be influenced by PPIs, considering the effects of PPIs on neutrophils or arteries. The data presented here suggest that the new activities of PPIs should be evaluated in the light of their role as inhibitors of TCTP and in the export of unidentified proteins export via ER-Golgi independent secretion pathways.

Several possible models can link, at the molecular level, the known actions of PPIs, their effect on their target (the α1-subunit of H^+^/K^+^-ATPase) and their ability to inhibit TCTP export. For example, the translocation apparatus exporting TCTP from cells may be regulated via the electrochemical gradient maintained by H^+^/K^+^-ATPase. If this model is correct, one needs to examine the expression of H^+^/K^+^-ATPase in U937 and HEK293 cells. Western blotting analysis using the cell lysates of U937 and HEK293 cells, showed several bands detected by anti-gastric H^+^/K^+^-ATPase α antibody (data not shown). But the detected proteins were smaller than expected. This indicates either that gastric H^+^/K^+^-ATPase α does not exist in these cells or that new, modified forms of gastric H^+^/K^+^-ATPase α might exist. It is also possible that there is a novel PPIs-sensitive protein target that is distinct from the catalytic α-subunit of H^+^/K^+^-ATPase, even though thus far, the main binding target of PPIs is the catalytic α-subunit of H^+^/K^+^-ATPase. Next possibility is the H^+^/K^+^-ATPase blockade may modify ongoing intracellular events, such as calcium influx and calcium mobilization, and modulate TCTP release. In previous studies, it was reported that PPIs could affect the intracellular calcium level [28], [29] and tat exocytosis is one of the mechanisms of TCTP secretion [30]. The changes in intracellular calcium levels caused by PPIs might influence exocytosis and subsequently TCTP secretion.

TCTP has been implicated in various cellular processes, such as cell growth, cell cycle progression, malignant transformation, protection of cells against stress and apoptosis. In addition, an extracellular, cytokine-like functions have been established for TCTP. Thus far, there have been no reports showing that changes in intracellular TCTP levels influence the severity of allergic responses or regulation of various allergic cytokines. For TCTP to act as a regulator of allergic responses, it should be released from cells. TCTP cannot affect downstream effector cells until it is secreted. Because of their ability to inhibit TCTP secretion *in vitro* and *in vivo*, omeprazole and pantoprazole might be potentially useful as anti-allergic asthmatic therapies.
